# Associations of calligraphy practice with executive function and transfer: a cross-sectional comparison of professional calligraphers and beginners

**DOI:** 10.3389/fpsyg.2026.1932736

**Published:** 2026-10-02

**Authors:** Xiaoping Su

**Affiliations:** Kangwon National University, Chuncheon, Republic of Korea

**Keywords:** association, calligraphy practice, cross-sectional comparison, executive function, far transfer, near transfer

## Abstract

**Introduction:**

Calligraphy practice is a complex activity involving visuospatial analysis, fine-motor control, attention, and self-monitoring. This study examined cross-sectional associations among calligraphy-practice experience, executive-function (EF) performance, near- and far-transfer task performance, visuospatial structural processing (VSP), and selfregulation (SR).

**Methods:**

We compared 310 professional calligraphers with 310 beginners. Groups were defined using prespecified criteria for training duration, current practice, and professional background; the Calligraphy Proficiency Validation Score (CVS) was used only after grouping as an independent validation measure. After screening, 338 professionals and 312 beginners were eligible. Random subsampling in SPSS 26.0, conducted before outcome analysis and based only on group membership, produced two groups of 310. Adjusted cross-sectional models examined group differences, Practice–EF associations, near- versus fartransfer differences, a bootstrap indirect association through VSP, and a Practice × SR interaction.

**Results:**

Professionals had higher adjusted EF (*B* = 0.415), near-transfer (*B* = 0.548), and far-transfer (*B* = 0.248) scores than beginners. The between-group difference was larger for near than far transfer (Group × transfer type *B* = 0.300). Practice was positively associated with EF (*B* = 0.278). VSP showed a significant bootstrap indirect association between Practice and EF, and the Practice × SR term was statistically significant.

**Discussion:**

The findings describe cross-sectional associations and conditional statistical patterns rather than directional or causal effects. Longitudinal and intervention studies are needed to establish temporal ordering and causality.

## 1 Introduction

Calligraphy practice has been discussed in the context of art education, traditional culture inheritance and skill training for a long time, and its cognitive attribute still has room for further development (Chu, 2025; Han et al., 2024). As far as the practical process is concerned, calligraphy is not a simple repetition of strokes, but includes continuous links such as inscription observation, spatial proportion judgment, stippling control, structure comparison, movement adjustment and self-evaluation (Wang et al., 2023). Professional calligraphers form relatively stable writing strategies in long-term copying, creation and style transformation, while beginners are mostly in the stage of basic imitation and external guidance (Nozawa, 2025; Yuan et al., 2025). The differences between the two groups provide an analyzable empirical basis for investigating the relationship between calligraphy practice and executive function performance. Executive function is an important cognitive ability for individuals to allocate attention, suppress response, maintain information and change rules around task goals (Friedman and Robbins, 2022). Observation, comparison, error correction and structural arrangement in calligraphy practice are related to inhibition control, working memory, cognitive flexibility and executive attention (Gandotra et al., 2022). However, whether there is a stable relationship between long-term calligraphy experience and the performance of these executive functions, and whether its correlation is more concentrated on calligraphy related tasks, or can be extended to general cognitive tasks, still needs to be tested through empirical design (Chen et al., 2024; Hsiao et al., 2023). Therefore, based on the comparison between professional calligraphers and calligraphy beginners, this paper further distinguishes between near transfer and far transfer.

This paper examines calligraphy practice within an empirical framework of executive function and transfer, focusing on cross-sectional differences between professional calligraphers and beginners and on associations involving years of study, weekly practice frequency, duration per session, and breadth of calligraphy experience. VSP is examined as a possible indirect statistical correlate, and SR is examined through a Practice × SR interaction. These models are intended to clarify association patterns across calligraphy education, art-training, and cognitive research; they do not establish temporal direction or intervention benefit.

## 2 Literature review and research hypothesis

### 2.1 Cognitive training of calligraphy practice

Calligraphy practice has an obvious attribute of compound cognitive training, and its process is not only stroke repetition and skilled movement, but also composed of observation, comparison, control, adjustment and evaluation (Hajikarim-Hamedani et al., 2025). When copying tablets, beginners need to continuously identify the position, structural proportion and spatial relationship of stipples, and maintain attention in writing, suppress wrong actions, and correct the gesture according to the template feedback (Zhang, 2022). With the accumulation of practice experience, learners also need to convert rules between different calligraphy styles, inscriptions and creative situations to form a more stable way of structural processing and self-regulation (Han et al., 2024; Wang and Tang, 2024). Therefore, calligraphy practice can be understood as a long-term cognitive training process embedded with cultural aesthetics and movement control, which provides a theoretical basis for investigating the relationship between calligraphy practice and executive function and transfer pattern.

### 2.2 Learning differences between professional calligraphers and calligraphy beginners

The difference between professional calligraphers and calligraphy beginners is not only reflected in the writing years and skill proficiency, but also reflected in the differences in learning strategies, structural processing and self-regulation (Kong et al., 2025). Calligraphy beginners usually rely on model, teacher demonstration and partial imitation, and their learning focuses on the position of stippling, stroke specification and basic structure. Cognitive resources are mostly used to control actions and avoid errors (Huang and Qiao, 2024). Professional calligraphers form relatively stable visual judgment, spatial organization and writing decision-making ability in long-term copying, comparison, creation and style transformation, and can flexibly adjust between different inscriptions, calligraphy styles and expression situations (Nozawa, 2021, 2023). Thus, the two groups provide a clear comparative basis for analyzing the relationship between calligraphy practice experience and executive function performance.

### 2.3 Executive function and its plasticity

Executive function refers to the regulation of cognition and behavior around task goals and commonly includes inhibitory control, working memory, cognitive flexibility, and executive attention (Ferguson et al., 2021). These components are related to attention maintenance, information updating, response inhibition, and rule switching. Training responsiveness and generalization vary across components and designs (Bombonato et al., 2024; Scionti et al., 2020). Performance is more often shared across tasks with similar demands, whereas far transfer to general tasks is less consistent and depends on component overlap (Birtwistle et al., 2025). In this study, this literature motivates cross-sectional association tests rather than claims about within-person cognitive change.

### 2.4 Near transfer and far transfer

Transfer research examines whether performance patterns associated with one learning context are also observed in new task contexts (Gobet and Sala, 2023; Zelazo et al., 2024). In this study, near transfer refers to calligraphy-related tasks involving font structure, spatial proportion, stroke-direction conflict, and calligraphic-structure recognition (Xu and Shen, 2023). Far transfer refers to general cognitive tasks that do not use calligraphic content, including inhibition, working-memory, task-switching, and executive-attention tasks (Yue et al., 2023). The near/far distinction is treated as a difference in task similarity and observed association, not as evidence that calligraphy practice produced improvement. Similar processing demands may help explain why a stronger cross-sectional group difference could be observed for near than far transfer (Liu et al., 2022).

### 2.5 Research hypotheses

Based on the cognitive attributes of calligraphy practice, group differences in experience, executive-function theory, and the distinction between near and far transfer, the study specifies hypotheses about cross-sectional group differences, associations, indirect associations, and a statistical interaction (Figure 1).

**FIGURE 1:**
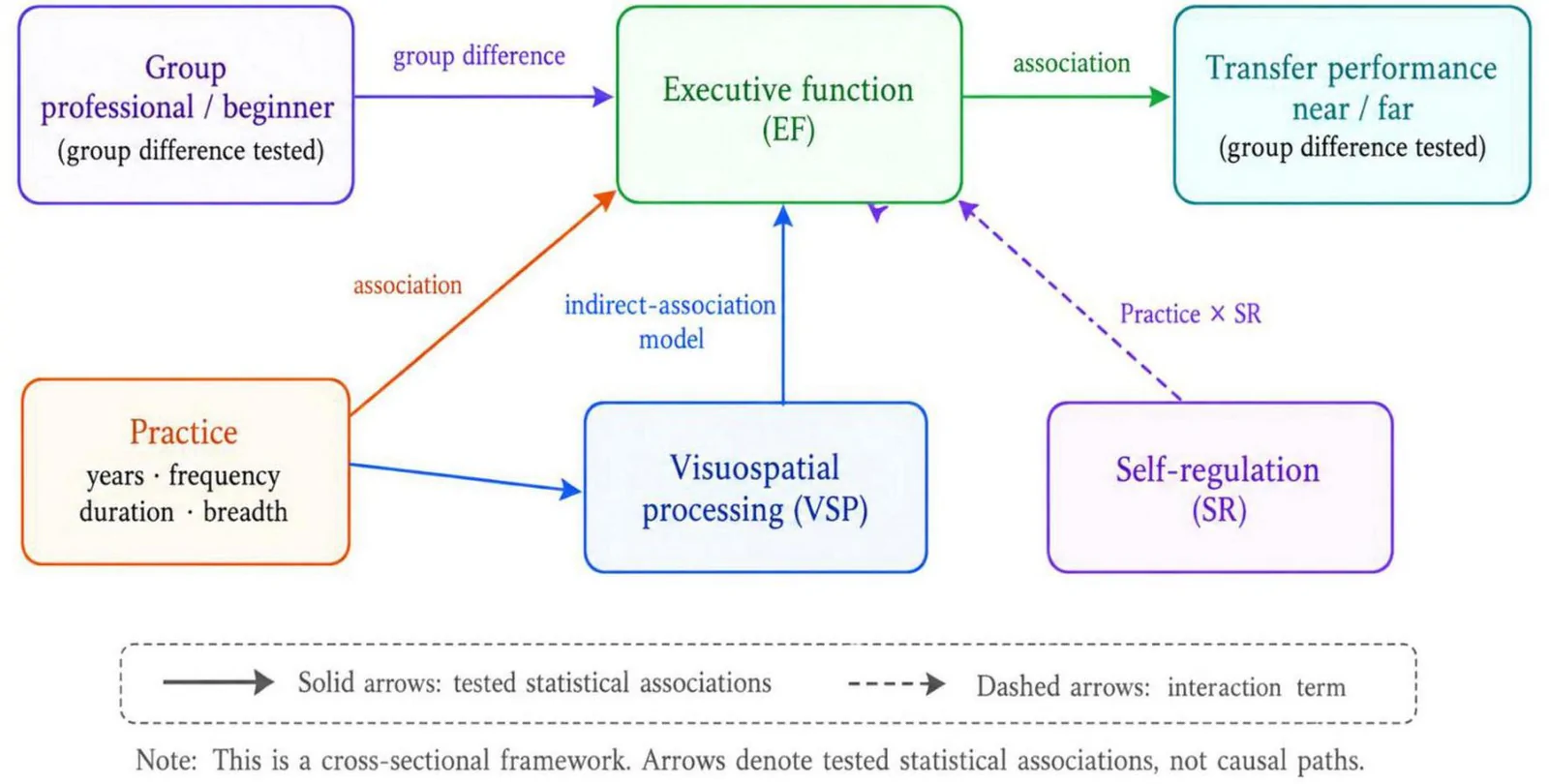
Cross-sectional conceptual framework (arrows denote statistical associations, not causal paths).

H1: Professional calligraphers will show higher executive-function composite scores than beginners. Long-term, stable, goal-directed experience is often associated with attention control, information maintenance, response inhibition, and rule switching (Luciana and Collins, 2022). H1 concerns a cross-sectional group difference and does not presume temporal direction.

H2: The professional–beginner difference will be larger for calligraphy-related near-transfer tasks than for general far-transfer tasks. Transfer accounts emphasize similarity in processing components across task contexts (Fransen, 2024). Accordingly, the hypothesis concerns a Group × transfer-type statistical interaction rather than within-person transfer change.

H3: Years of study, weekly practice frequency, duration per session, and breadth of calligraphy experience will be positively associated with executive-function performance. These indicators represent different aspects of accumulated practice experience (Hambrick et al., 2020); the hypothesis concerns covariance, not cognitive change over time.

H4: VSP will show a significant bootstrap indirect association between calligraphy practice and executive-function performance. Visuospatial accounts suggest that complex symbol learning is related to analysis of spatial proportion, local relations, and overall structure (Wei et al., 2024). In the present cross-sectional data, the indirect term is interpreted as an explanatory association pattern and not as a time-ordered process.

H5: SR will statistically moderate the association between calligraphy practice and executive-function performance, operationalized by the Practice × SR interaction. Self-regulated-learning theory links goal setting, process monitoring, error correction, and outcome evaluation with how learners organize practice experience (Huang et al., 2024). This hypothesis concerns variation in a conditional association and not a directional amplification process.

## 3 Research design

### 3.1 Participants, grouping, tasks, and procedure

This study used a cross-sectional comparison of professional calligraphers and calligraphy beginners. Recruitment sources included university calligraphy- and art-related programs, university calligraphy associations, public art-education courses, social calligraphy-training institutions, and calligraphy-practice platforms. Eligible recruits were aged 18 years or older, had normal or corrected-to-normal vision, and reported no neurological disorder, psychiatric disorder, or injury affecting fine hand movements. The analytic sample ranged from 18 to 28 years of age.

#### 3.1.1 Prespecified operational group definitions

**Professional Calligrapher Group.** Participants had to satisfy all of the following: (a) at least 5 years of formal calligraphy training with a professional teacher or in a formal course; (b) current practice at least three times per week and at least 60 min per session; and (c) at least one professional-background criterion: membership in a municipal-level or higher calligraphers’ association, at least 2 years of calligraphy-teaching experience, selection of work for a municipal-level or higher exhibition or competition, or current/completed undergraduate-or-higher study in a calligraphy major. Classification was based on the screening questionnaire and available documentary evidence, including membership, teaching, exhibition, or educational records.

**Calligraphy Beginner Group.** Participants had to report no formal calligraphy training or no more than 3 months of cumulative training, no sustained current practice (no more than once per week), and no professional certification, teaching, competition, or exhibition experience. Classification was based on the screening questionnaire.

**Role of CVS.** CVS was not used to assign participants to groups. It was administered only after classification as an independent group-validation measure. Group assignment was based exclusively on training duration, current practice frequency, and professional-background criteria.

**Experience-consistency screening.** The reported age at starting calligraphy, total years of training, weekly practice frequency, and current-practice status were cross-checked. A record was flagged if total training was at least 3 years but current practice was 0 times/week and no creation/competition experience was reported, or if starting age plus total training differed from current age by more than 2 years without a documented interruption. Flagged participants were contacted by telephone or online for clarification. Unresolved cases were excluded. Two research assistants judged each case independently; disagreement was resolved by a third researcher.

**Other long-term training.** Prespecified potential competing training included professional music, painting, dance, and fine-motor sports such as table tennis, shooting, or archery. Participants were excluded if they had at least three consecutive years of professional training in any listed activity and were still practicing more than 2 h per week. Training discontinued for more than 2 years was retained and coded as past training.

**Sample balancing and selection.** The target of 310 per group was achieved by random subsampling, not propensity-score matching or outcome-based selection. The procedure used SPSS 26.0 with seed 20240115 and was completed before primary analyses. It selected 310 of 338 eligible professionals and 310 of 312 eligible beginners solely from the group indicator.

As shown in Figure 2 and Table 1, 870 individuals were recruited. The six reason-specific screening counts are non-mutually exclusive because one person could trigger more than one flag; they must not be subtracted sequentially. After de-duplication, 220 unique individuals were excluded and 650 were eligible (338 professionals and 312 beginners). Before any outcome analysis, random subsampling was performed in SPSS 26.0 with seed 20240115 and using group membership only: 28 professional records and 2 beginner records were not selected, yielding 310 participants in each group (*N* = 620). No matching on outcomes, CVS, EF, NT, FT, VSP, SR, or covariates was performed.

**FIGURE 2:**
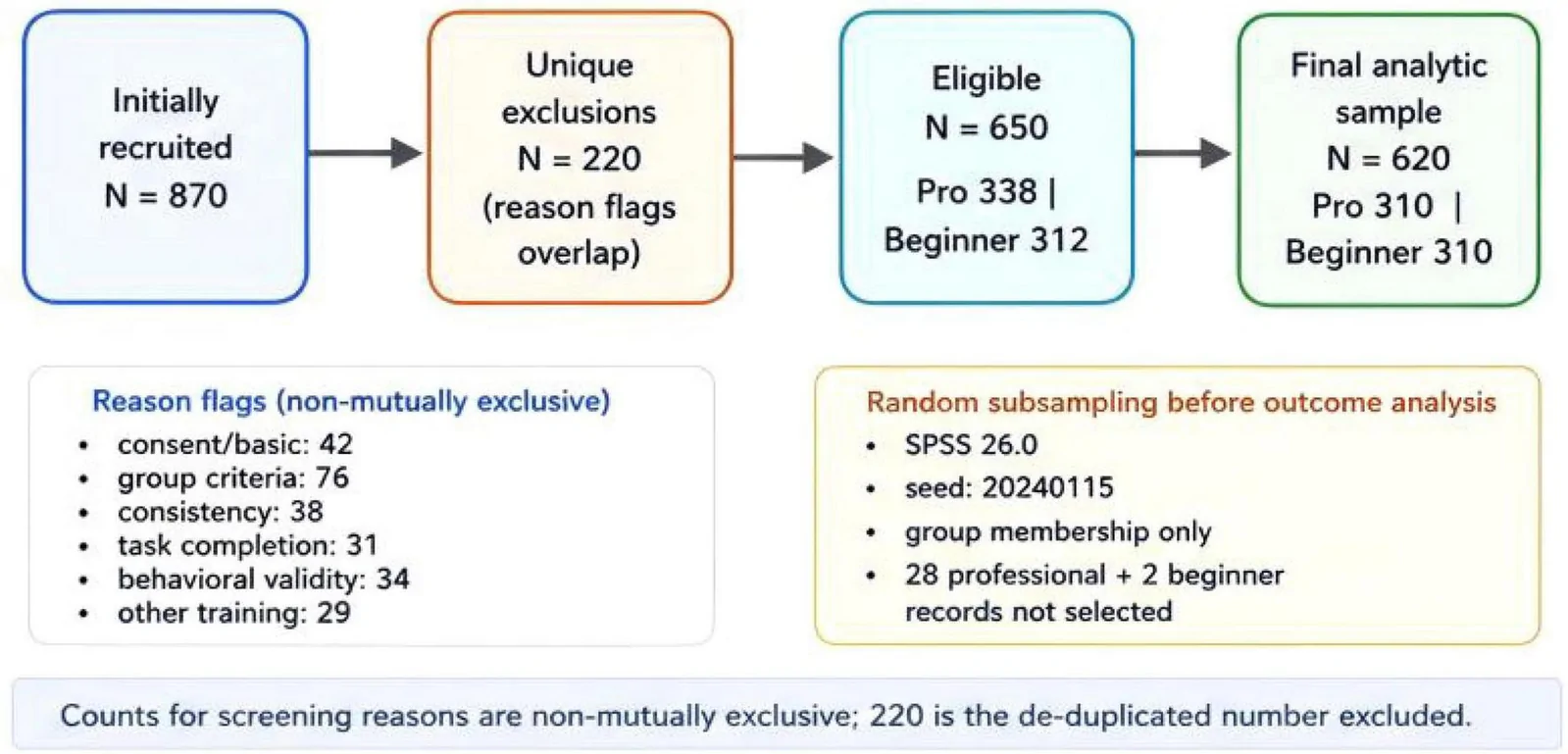
Sample screening, de-duplication, and random subsampling.

**TABLE 1:** Research sample screening and analytic sample composition.

Stage	Criterion or operation	Count	Sample status
Initial recruitment	Recruitment sources described in Section 3.1	870	870 recruited
Consent/basic-information flag	Unconfirmed consent or missing demographic information	42	Reason-specific; may overlap
Group-criteria flag	Did not satisfy either operational group definition	76	Reason-specific; may overlap
Experience-consistency flag	Unresolved inconsistency after cross-check and clarification	38	Reason-specific; may overlap
Task-completion flag	More than 10% of required behavioral-task data missing	31	Reason-specific; may overlap
Behavioral-validity flag	EF accuracy below 60% or more than 20% invalid RT trials in any EF task	34	Reason-specific; may overlap
Other-training flag	Current competing professional training met the prespecified threshold	29	Reason-specific; may overlap
Unique excluded after screening	De-duplicated individuals meeting at least one exclusion rule	220	650 eligible
Eligible before balancing	Professional = 338; beginner = 312	650	338/312
Random subsampling	SPSS 26.0; seed 20240115; group-only selection; professional 28 and beginner 2 not selected	30	310/310
Final analytic sample	Professional = 310; beginner = 310	620	*N* = 620

The procedure comprised four stages: (1) demographic, calligraphy-experience, and other-training questionnaires; (2) post-group CVS assessment, including prescribed-copy performance, font-structure judgment, and expert blind rating; (3) calligraphy-related near-transfer and general-cognitive far-transfer tasks; and (4) a separate VSP task, EF tasks, and the SR scale. Task order was randomized. All participants provided informed consent, and the study received relevant ethics review. The ethics committee name, approval number, and approval date should be inserted before submission.

The revised sample flow distinguishes unique exclusions from reason-specific flags and later balancing. Table 1 shows that 870 individuals were recruited, 220 unique cases were excluded, and 650 participants remained eligible. The eligible pool contained 338 professionals and 312 beginners. Random subsampling then produced an analytic sample of 620, with 310 participants retained in each group. This sequence explains the equal final groups without implying matching on outcomes or covariates. Because a participant could meet more than one screening condition, the reason-specific counts are best interpreted as overlapping audit flags. The reported flow is coherent provided that the de-duplicated exclusion count and random-selection log are supported by the original records.

#### 3.1.2 Cognitive-task implementation and response-time preprocessing

**Author verification required before submission.** The files supplied for revision do not contain the archived task scripts or log-file metadata needed to verify task versions, exact trial counts, stimulus lists, trial timing, or acquisition software. Appendix Table 1 therefore identifies every field that must be copied from the original scripts. These highlighted verification items must not remain unresolved in the submitted manuscript.

**Reaction-time preprocessing.** RT analyses used correct trials only. Trials faster than 150 ms or slower than 3,000 ms were removed. A participant failed behavioral-task validity screening if more than 20% of trials were removed in any EF task; the participant’s record was then excluded from the analytic sample. These trial- and participant-level rules were prespecified and applied during preprocessing before the primary analyses. The separate completion rule excluded records with more than 10% missing required behavioral-task data, and EF task accuracy below 60% was an additional participant-level validity criterion.

#### 3.1.3 Construct separation and required item-level audit

Calligraphy Proficiency Validation Score, NT, VSP, EF, and FT must be calculated from mutually exclusive source items, trials, and score columns. Conceptual similarity is acceptable, but literal reuse is not. The available manuscript and summary tables establish that CVS is excluded from the Practice composite, but they do not provide item-level identifiers sufficient to prove separation for all behavioral constructs. Appendix Table 2 records the audit status and the required action.

**Reanalysis rule.** If the audit identifies any reused item, trial, or derived score, construct non-overlapping composites from the raw trial-level data and rerun descriptive statistics, correlations, group-validation tests, all regression and mixed models, bootstrap indirect-association analysis, moderation analysis, and robustness checks. Numerical results should not be represented as final until this audit is complete.

### 3.2 Measurement of variables related to calligraphy practice

This study specified measures of calligraphy-practice level, EF, near transfer, far transfer, VSP, and SR. Questionnaire variables used seven-point response scales. Behavioral indicators were derived from prespecified accuracy and/or reaction-time scores and transformed so that higher values represented better performance. Practice combined standardized years of study, weekly frequency, duration per session, and breadth of experience; CVS was not included in that composite. Exact scoring equations, weights, reliability indices, and source-column identifiers must be reported from the archived scoring code.

As shown in Table 2, EF comprises inhibitory control, working memory, cognitive flexibility, and executive attention. NT comprises calligraphy-specific tasks, whereas FT comprises general cognitive tasks. VSP, EF, NT, FT, and CVS must use separate source tasks or mutually exclusive item/trial sets. All behavioral indicators were direction-aligned so higher scores denoted better performance; the exact task-level formulas and composite weights require confirmation from the archived code (Appendix Tables 1, 2).

**TABLE 2:** Summary of variables.

Variable type	Variable name	Abbreviation	Definition and measurement method
Independent variable	Group variable	Group	The professional calligrapher group is assigned a value of 1, and the calligraphy beginner group is assigned a value of 0, which is used to distinguish groups with different calligraphy practice levels.
	Comprehensive level of calligraphy practice	Practice	It is synthesized after standardization of calligraphy study years, weekly practice frequency, single practice duration and calligraphy experience breadth.
Grouping validation variables	Calligraphy proficiency verification score	CVS	Post-group validation only: prescribed copying, font-structure judgment, and expert blind rating. CVS did not determine group assignment and is not part of Practice.
Dependent variable	Comprehensive performance of executive function	EF	Composite of inhibitory control, working memory, cognitive flexibility, and executive attention from independent source tasks; no FT score may be reused.
Transfer variable	Near-transfer task performance	NT	Composite of calligraphy-specific tasks using item/trial sets that are disjoint from CVS and VSP.
	Far-transfer task performance	FT	Composite of general cognitive tasks using source tasks and score columns that are disjoint from EF.
Indirect-association variable	Visual spatial structure processing ability	VSP	Separately administered visuospatial measure; no CVS or NT item, trial, or score may be reused.
Moderator	Self-regulation ability	SR	Self-regulation scale used in the Practice × SR statistical interaction.
Control variable	Age	Age	The actual age of the subjects shall be recorded according to the first year of life.
	Gender	Gender	The male is assigned a value of 1 and the female is assigned a value of 0. Other cases are coded according to the actual situation.
	educational level	Edu	Subjects’ current or completed highest education stage shall be treated as orderly classified variables.
	Dominant hand	Hand	The right hander is assigned a value of 1, and the left hander or double hander is assigned a value of 0.
	General intelligence	IQ	Obtained through the short version reasoning test or the standardized cognitive ability test to control the differences in general cognitive ability.
	Other training experience	OT	The subjects’ experience of art training or sports training other than calligraphy is recorded by years or grades.
	Frequency of use of digital equipment	DU	The frequency of subjects’ daily use of digital devices such as mobile phones, computers and tablets was measured with the seven-point Likert scale.

VSP was modeled as an intermediate correlate in the bootstrap indirect-association analysis. SR was modeled as a moderator through the Practice × SR product term. Control variables were age, gender, educational level, handedness, general intelligence, other training experience, and digital-device use. Scale reliability and behavioral-task reliability should be reported for each measure; these indices cannot be reconstructed from the summary statistics alone (Figure 3).

**FIGURE 3:**
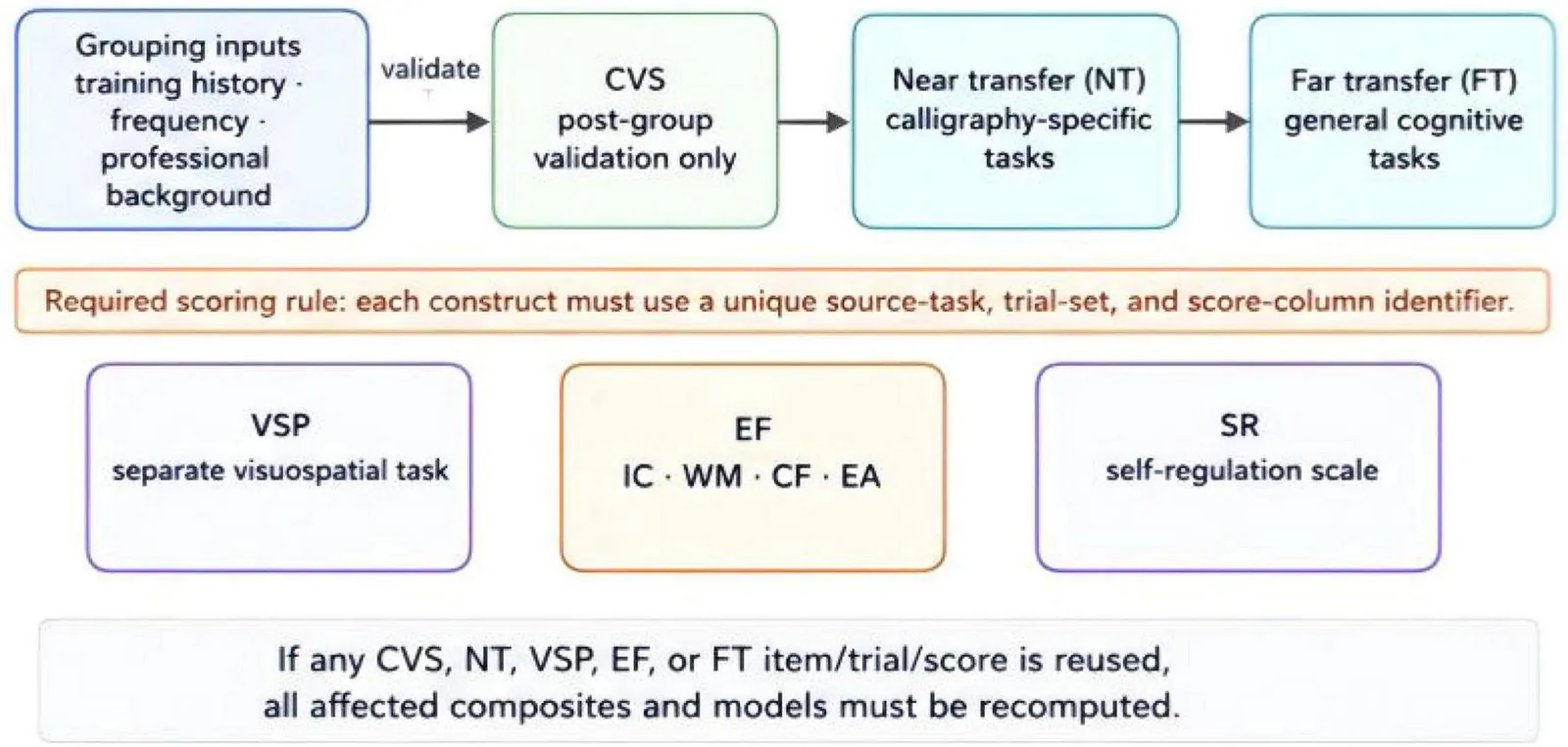
Measurement structure and required separation of task items, trials, and scores.

The variable system combines group classification, practice exposure, cognitive performance, transfer, and covariate adjustment in one framework. Table 2 lists 15 variables, codes professionals as 1 and beginners as 0, forms Practice from 4 indicators, and represents EF through four components. SR uses a seven-point response scale, while the model includes seven prespecified covariates. This organization clarifies the role of each measure and reduces ambiguity between grouping, validation, outcomes, and adjustment variables. The separation of CVS from Practice is especially important because it preserves CVS as an external validation measure. The framework remains interpretable only if the behavioral composites are calculated from independent source tasks and fully documented scoring rules.

### 3.3 Cross-sectional association and transfer models

The statistical models were specified around calligraphy-practice level, transfer-task type, VSP, and SR. They estimate adjusted cross-sectional group differences, Practice–EF associations, differences between near and far transfer, a bootstrap indirect association, and a Practice × SR statistical interaction.

To estimate cross-sectional differences between professional calligraphers and beginners and the adjusted association between Practice and EF, the first model family regressed EF on either Group or Practice and the prespecified covariates. Coefficients are interpreted as adjusted differences or associations, not directional estimates of practice-related change.


$$E\,F_{i}=\beta_{0}+\beta_{1}\,C\,a\,l\,l\,i\,g\,r\,a\,p\,h\,y_{i}+\beta_{2}\,C\,o\,n\,t\,r\,o\,l\,s_{i}+\varepsilon_{i}$$ (1)

In Equation 1, *EF*_*i*_ denotes participant i’s executive function composite; *Calligraphy*_*i*_ denotes either group status or the comprehensive calligraphy practice score; *Controls*_*i*_ denotes age, gender, education, handedness, general intelligence, other training experience, and digital-device use; β_0_ is the intercept; β_1_ is the focal group difference or practice association coefficient; β_2_ denotes the coefficients for the control variables; and ε_*i*_ is the residual.

To compare near-transfer and far-transfer performance, the second model family included Group, transfer type, and their statistical interaction. The Group × transfer-type term tests whether the professional–beginner difference varies across near and far transfer; it does not estimate change produced by practice.


$$P\,e\,r\,f\,o\,r\,m\,a\,n\,c\,e_{i\,j}=\beta_{0}+\beta_{1}\,G\,r\,o\,u\,p_{i}+\beta_{2}\,T\,r\,a\,n\,s\,f\,e\,r\,T\,y\,p\,e_{j}$$ (2)

$$+\beta_{3}\,G\,r\,o\,u\,p_{i}\times T\,r\,a\,n\,s\,f\,e\,r\,T\,y\,p\,e_{j}+\beta_{4}\,C\,o\,n\,t\,r\,o\,l\,s_{i}+u_{i}+\varepsilon_{i\,j}$$

In Equation 2, *Performance*_*ij*_ denotes participant i’s performance for transfer type j; Group_*i*_ is coded 1 for professional calligraphers and 0 for beginners; Transfer Type_*j*_ is coded 1 for near transfer and 0 for far transfer; Group_*i*_ × Transfer Type_*j*_ is the interaction term; Controls_*i*_ check if the captured denotes the prespecified covariates; u_*i*_ is the participant-specific random intercept; and ε_*ij*_ is the residual.

The third model family examined (a) a bootstrap indirect association linking Practice, VSP, and EF and (b) a Practice × SR statistical interaction. Because all variables were measured cross-sectionally, the indirect term is not interpreted as a time-ordered pathway, and the interaction is not interpreted as evidence that SR changes an intervention response.


$$V\,S\,P_{i}=\alpha_{0}+\alpha_{1}\,P\,r\,a\,c\,t\,i\,c\,e_{i}+\alpha_{2}\,C\,o\,n\,t\,r\,o\,l\,s_{i}+\mu_{i}$$ (3)

$$E\,F_{i}=\gamma_{0}+\gamma_{1}\,P\,r\,a\,c\,t\,i\,c\,e_{i}+\gamma_{2}\,V\,S\,P_{i}+\gamma_{3}\,S\,R_{i}$$

$$+\gamma_{4}\,P\,r\,a\,c\,t\,i\,c\,e_{i}\times S\,R_{i}+\gamma_{5}\,C\,o\,n\,t\,r\,o\,l\,s_{i}+v_{i}$$

In Equation 3, the terms represent Practice, VSP, SR, the Practice × SR interaction, covariates, and the residual. The models first estimate the Practice–VSP association and then the conditional Practice–EF association. Bootstrap confidence intervals describe an indirect association; they do not establish temporal ordering.

Together, the three model families address cross-sectional group comparison and Practice–EF association, near/far transfer differences, and potential explanatory association patterns. Figure 4 summarizes the statistical architecture. The interpretation throughout is associational because exposure and outcomes were not experimentally assigned or measured longitudinally.

**FIGURE 4:**
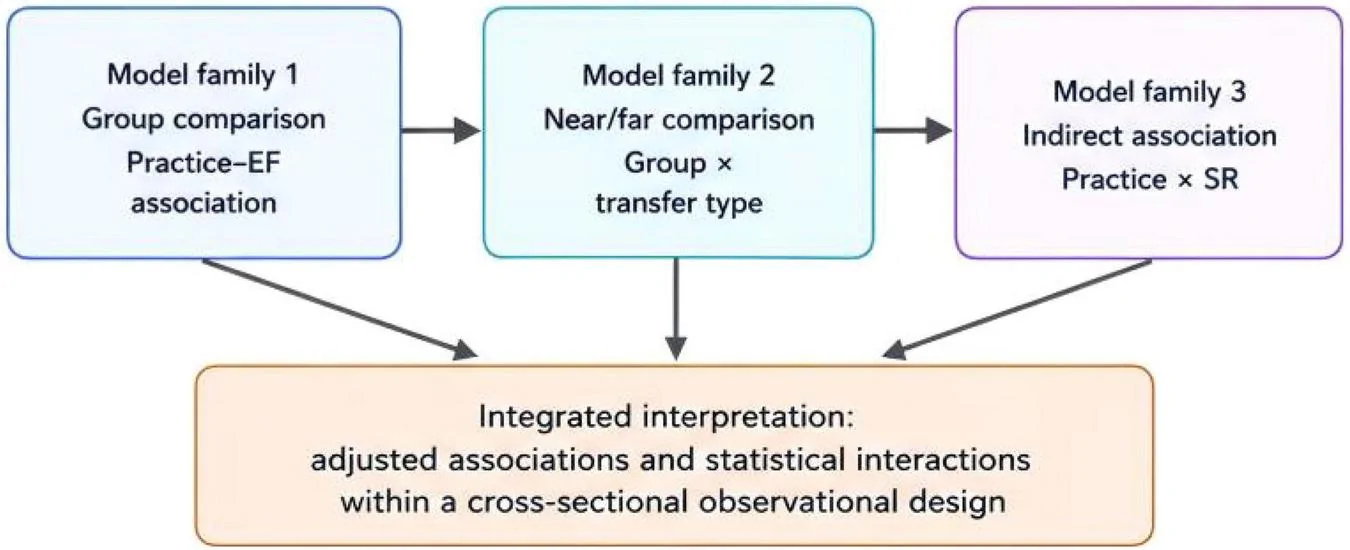
Cross-sectional statistical model architecture.

## 4 Empirical analysis

### 4.1 Descriptive statistics, distribution test, and correlation analysis

Descriptive patterns show substantial separation between the professional and beginner groups across practice and cognitive measures. Table 3 reports Practice means of 1.02 and −0.98, EF means of 0.43 and −0.41, near-transfer means of 0.46 and −0.40, and far-transfer means of 0.21 and −0.25 for professionals and beginners, respectively.

**TABLE 3:** Descriptive statistics and distribution tests by group.

Variable	Abbrev.	Full *M*	Full SD	Professional *M*	Professional SD	Beginner *M*	Beginner SD	Skew.	Kurt.	K–S Z	K–S *p*
Comprehensive calligraphy practice score	Practice	0.02	1.291	1.02	0.85	−0.98	0.78	−0.15	0.08	0.84	0.48
Calligraphy proficiency validation score	CVS	51.3	21.21	68.48	11.24	34.12	13.52	−0.11	−0.87	0.98	0.29
Executive function composite	EF	0.01	1.02	0.43	0.94	−0.41	0.93	0.06	−0.13	0.82	0.52
Near-transfer task performance	NT	0.03	1.002	0.46	0.91	−0.40	0.91	−0.02	0.11	0.79	0.56
Far-transfer task performance	FT	−0.02	1.013	0.21	0.97	−0.25	1.01	0.09	−0.08	0.93	0.35
Visuospatial structural processing	VSP	0	1.009	0.37	0.93	−0.37	0.94	0.01	0.03	0.77	0.59
Self-regulation	SR	4.65	1.186	5.03	1.06	4.27	1.19	−0.34	0.22	1.18	0.12
Inhibitory control	IC	0	1.01	0.34	0.94	−0.34	0.97	0.05	−0.09	0.81	0.51
Working memory	WM	0	1.009	0.37	0.94	−0.37	0.95	0.02	−0.06	0.83	0.46
Cognitive flexibility	CF	0	1.01	0.31	0.95	−0.31	0.98	0.07	−0.10	0.85	0.4
Executive attention	EA	0	1.008	0.27	0.96	−0.27	0.99	0.04	−0.11	0.86	0.37
Age	Age	21.5	2.48	22.58	2.21	20.42	2.28	0.48	0.12	1.1	0.18
General intelligence	IQ	102.35	14.52	105.18	13.76	99.52	14.51	−0.13	0.06	0.92	0.36
Other training experience	OT	1.22	1.48	1.31	1.58	1.13	1.37	1.18	1.47	1.62	0.03
Digital-device use frequency	DU	4.48	1.44	4.36	1.41	4.6	1.46	−0.25	0.16	1.13	0.15

*N* = 620 (professional *n* = 310; beginner *n* = 310). Full-sample SDs use *N*−1; within-group variances use *n*−1 = 309. Practice is a composite of four standardized practice indicators and was not re-standardized in the full sample. K–S tests use the Lilliefors correction; the OT *p*-value is based on the corresponding corrected critical value.

Figure 5 provides a compact visual comparison of standardized practice and cognitive performance across the professional and beginner groups. The largest separation appears for the Practice composite, with professionals centered at 1.02 and beginners at −0.98, confirming that the operational grouping captures markedly different experience profiles. The same directional pattern is visible for EF and all four EF components: professionals have positive standardized means for EF, inhibitory control, working memory, cognitive flexibility, and executive attention, whereas beginners have negative means on each measure. Near-transfer performance also shows a clear separation (0.46 versus −0.40), while the far-transfer contrast is smaller (0.21 versus −0.25). VSP follows the broader cognitive pattern, with means of 0.37 and −0.37. The error bars indicate substantial within-group variability, so the figure should not be read as implying complete separation between individuals. Instead, it summarizes differences in group-level distributions that are later evaluated with adjusted models. Overall, the visual pattern is consistent with stronger professional–beginner differences for practice-proximal measures than for far-transfer performance, while the cross-sectional design means that these differences represent associations with group status rather than evidence of training-induced change.

**FIGURE 5:**
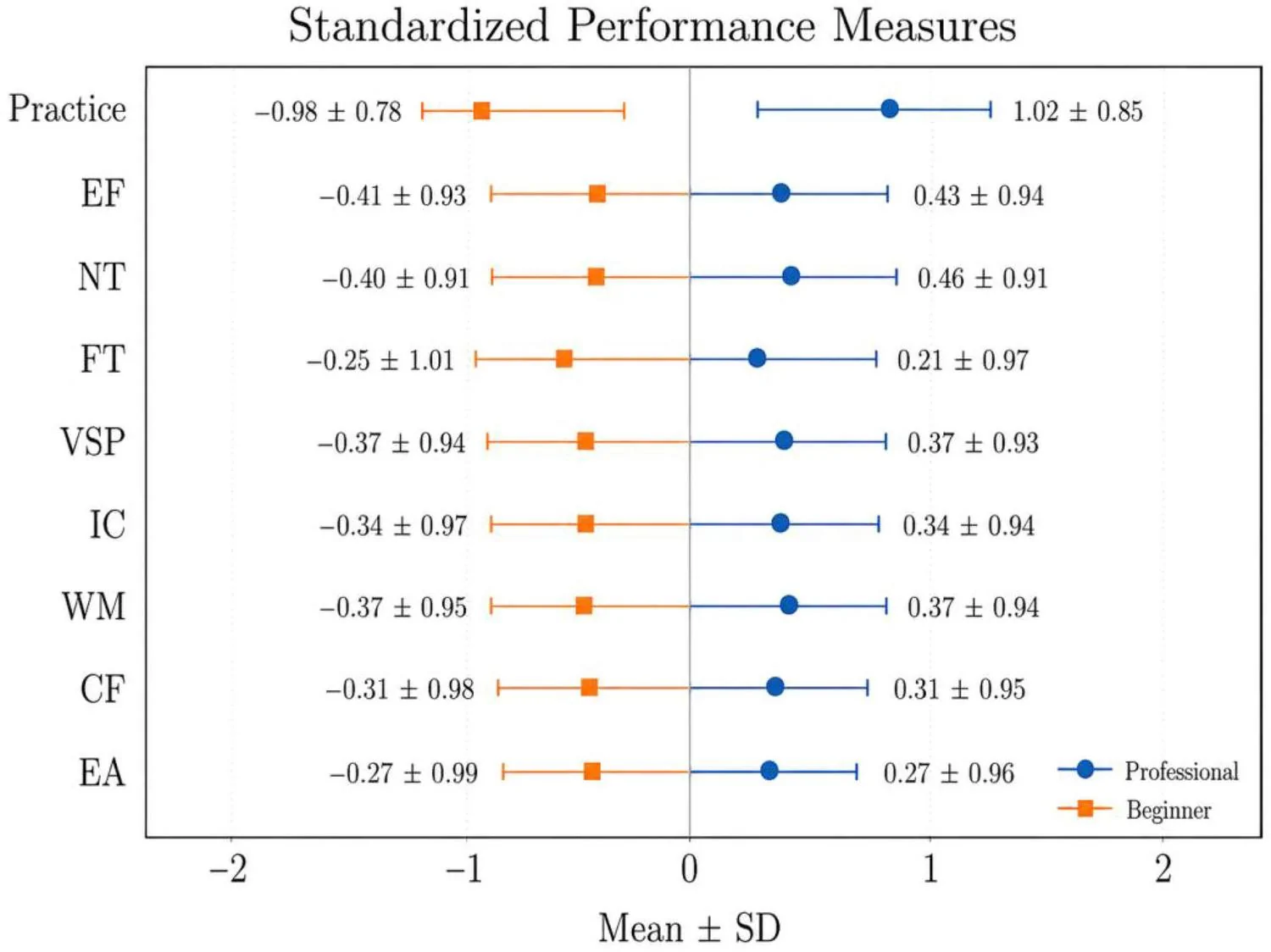
Standardized performance measures.

The direction is consistent across these domains, although the far-transfer contrast appears smaller than the near-transfer contrast. This pattern accords with the proposed distinction between task-proximal and more general performance. Because the statistics are cross-sectional and unadjusted, they describe observed group distributions rather than changes attributable to training. Multivariable models are therefore needed to assess whether the pattern remains after covariate adjustment.

Figure 6 condenses the strongest positive relationships among the study variables into a network, making the structure of the correlation matrix easier to interpret. Practice is strongly connected with CVS (*r* = 0.78), which is expected because CVS was designed as an independent validation measure of calligraphy proficiency. Practice is also linked with near transfer (*r* = 0.42) and VSP (*r* = 0.38), indicating that greater practice exposure co-occurs with stronger performance on task-proximal and visuospatial measures. EF occupies a central position in the network and is strongly related to its component scores, particularly working memory (*r* = 0.84), inhibitory control (*r* = 0.81), cognitive flexibility (*r* = 0.80), and executive attention (*r* = 0.78). Near transfer is connected with EF (*r* = 0.45), VSP (*r* = 0.40), and working memory (*r* = 0.39), which visually supports the idea of shared processing demands across related tasks. The figure displays only the top 19 positive correlations, all with *p* < 0.001; therefore, variables shown without edges should not be interpreted as unrelated. The network is descriptive and does not establish causal direction or temporal ordering among practice, proficiency, and cognitive performance.

**FIGURE 6:**
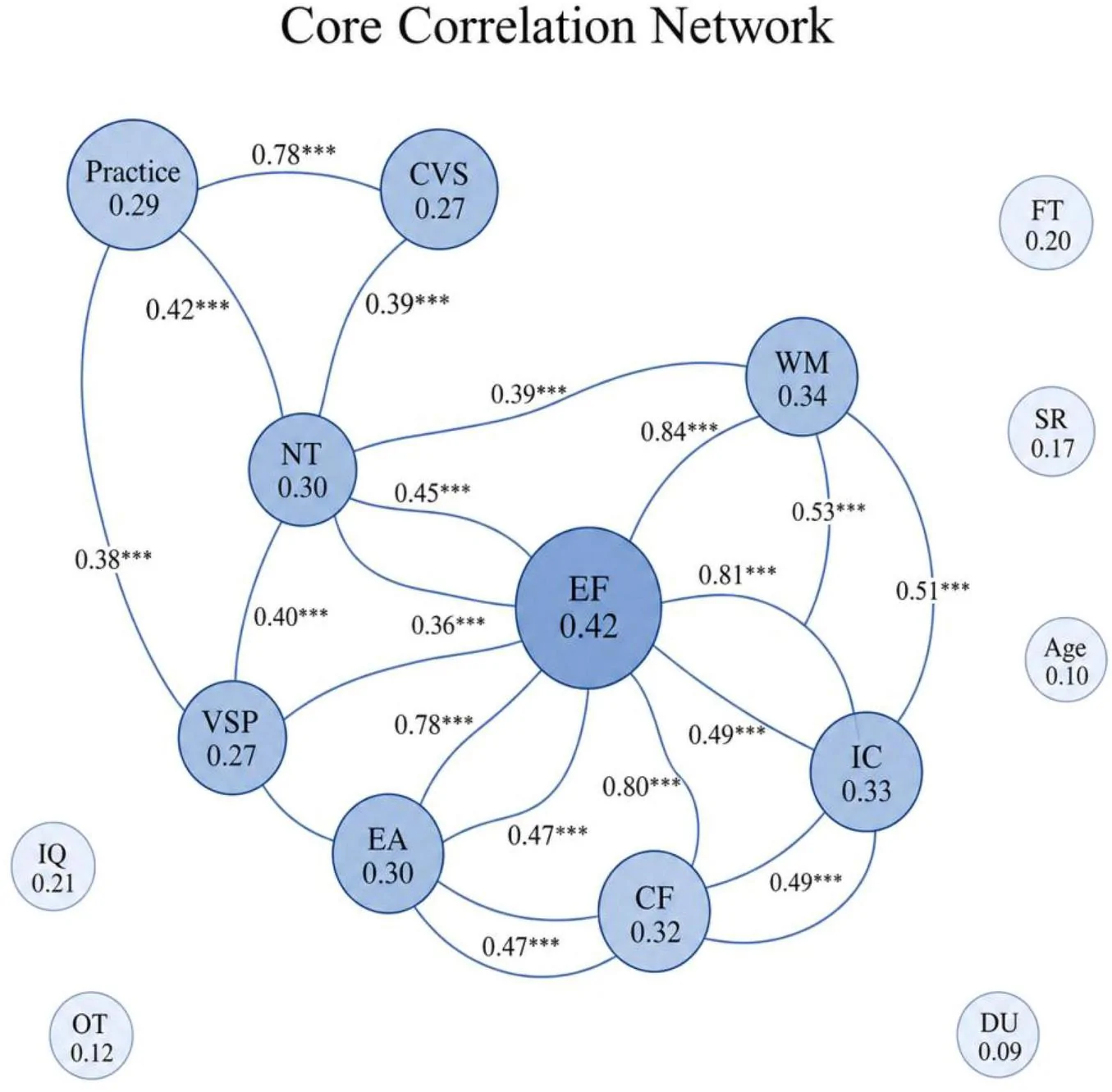
Core correlation network. Top 19 positive correlations are displayed; all shown links ***p < 0.001.

The correlation structure indicates that the study variables are related without being interchangeable. Table 4 shows that Practice correlates 0.78 with CVS and 0.35 with EF, while EF correlates 0.45 with near transfer and 0.28 with far transfer. VSP correlates 0.40 with near transfer, and IQ correlates 0.32 with EF. The stronger Practice–CVS relation is consistent with the intended validation role of CVS, whereas the more moderate cognitive correlations suggest distinguishable constructs. The larger EF relation with near than far transfer also fits the task-similarity account. These coefficients remain descriptive associations and should not be interpreted as evidence of temporal direction, especially because common background characteristics may contribute to several relationships.

**TABLE 4:** Extended correlation matrix.

Variable	1	2	3	4	5	6	7	8	9	10	11	12	13	14	15
1. Practice	1	–	–	–	–	–	–	–	–	–	–	–	–	–	–
2. CVS	0.78***	1	–	–	–	–	–	–	–	–	–	–	–	–	–
3. EF	0.35***	0.32***	1	–	–	–	–	–	–	–	–	–	–	–	–
4. NT	0.42***	0.39***	0.45***	1	–	–	–	–	–	–	–	–	–	–	–
5. FT	0.21***	0.18***	0.28***	0.25***	1	–	–	–	–	–	–	–	–	–	–
6. VSP	0.38***	0.34***	0.36***	0.40***	0.22***	1	–	–	–	–	–	–	–	–	–
7. SR	0.25***	0.22***	0.23***	0.19***	0.17***	0.20***	1	–	–	–	–	–	–	–	–
8. IC	0.30***	0.27***	0.81***	0.37***	0.22***	0.31***	0.19***	1	–	–	–	–	–	–	–
9. WM	0.33***	0.30***	0.84***	0.39***	0.24***	0.33***	0.21***	0.53***	1	–	–	–	–	–	–
10. CF	0.28***	0.25***	0.80***	0.35***	0.23***	0.30***	0.18***	0.49***	0.51***	1	–	–	–	–	–
11. EA	0.25***	0.22***	0.78***	0.34***	0.21***	0.28***	0.17***	0.47***	0.49***	0.47***	1	–	–	–	–
12. Age	0.18***	0.16***	0.12**	0.14***	0.08*	0.11**	0.08*	0.10*	0.11**	0.10*	0.09*	1	–	–	–
13. IQ	0.15***	0.13**	0.32***	0.24***	0.26***	0.28***	0.19***	0.27***	0.29***	0.26***	0.25***	0.07	1	–	–
14. OT	0.12**	0.10*	0.16***	0.13**	0.14***	0.12**	0.09*	0.14***	0.15***	0.13**	0.12**	0.06	0.12**	1	–
15. DU	−0.12**	−0.10*	−0.11**	−0.09*	−0.08*	−0.13**	−0.07	−0.09*	−0.09*	−0.08*	−0.10*	0.05	−0.06	0.04	1

***P < 0.001, **p < 0.01, *p < 0.05 (two-sided; *N* = 620). Stars are based on unrounded *p*-values. A supplementary Benjamini–Hochberg procedure (q = 0.05) was applied to the 105 unique correlations using unrounded *p*-values; no rounded critical *p*-value or retained-test count is reported here.

### 4.2 Grouping validation based on calligraphy practice measures

Group validation is supported by large observed differences in both practice exposure and independently assessed proficiency. Table 5 reports Practice means of 1.02 for professionals and −0.98 for beginners, with a t value of 30.52 and a Cohen d of 2.45. CVS means were 68.48 and 34.12, accompanied by a t value of 34.41 and a Cohen d of 2.76.

**TABLE 5:** Group differences in calligraphy practice and validation scores.

Measure	Professional M ± SD	Beginner M ± SD	Levene F	Levene *p*	Mean difference	t	df	*P*	Cohen’s *d*
Practice	1.02 ± 0.85	−0.98 ± 0.78	1.23	0.268	2	30.52	618	<0.001	2.45
CVS	68.48 ± 11.24	34.12 ± 13.52	2.15	0.143	34.36	34.41	618	<0.001	2.76

The convergence of a history-based practice index and a post-group proficiency measure strengthens confidence that the operational criteria distinguished markedly different experience profiles. This evidence validates the grouping procedure at the descriptive level, but it does not show that group membership itself produced later cognitive differences.

### 4.3 Executive-function and transfer-task group differences

Adjusted regression estimates suggest that group status and practice intensity remain related to EF after the listed covariates are considered. Table 6 shows an unadjusted Group coefficient of 0.840, which decreases to 0.415 in the covariate-adjusted model. IQ contributes 0.014 per raw-score unit, while the alternative Practice model yields a coefficient of 0.278 and a standardized coefficient of 0.352.

**TABLE 6:** Regression models for executive function.

Model	Outcome	Predictor	B	SE	β	t	*P*	95% CI
M1	EF	Group	0.84	0.076	0.411	11.05	<0.001	[0.691, 0.989]
		Constant	−0.410	0.054	–	−7.59	<0.001	[−0.516, −0.304]
M2	EF	Group	0.415	0.081	0.203	5.12	<0.001	[0.256, 0.574]
		Age	0.019	0.012	0.046	1.64	0.102	[−0.004, 0.042]
		Gender	−0.048	0.062	−0.023	−0.77	0.441	[−0.170, 0.074]
		Edu	0.082	0.051	0.04	1.61	0.108	[−0.018, 0.182]
		Hand	0.028	0.072	0.014	0.39	0.697	[−0.113, 0.169]
		IQ	0.014	0.003	0.199	4.88	<0.001	[0.008, 0.020]
		OT	0.033	0.021	0.048	1.57	0.117	[−0.008, 0.074]
		DU	−0.027	0.022	−0.038	−1.23	0.219	[−0.070, 0.016]
		Constant	−0.485	0.205	–	−2.37	0.018	[−0.888, −0.082]
M3	EF	Practice	0.278	0.048	0.352	5.79	<0.001	[0.184, 0.372]
		Age	0.019	0.012	0.046	1.63	0.103	[−0.004, 0.042]
		Gender	−0.047	0.062	−0.023	−0.76	0.448	[−0.169, 0.075]
		Edu	0.081	0.051	0.04	1.59	0.112	[−0.019, 0.181]
		Hand	0.027	0.072	0.013	0.38	0.704	[−0.114, 0.168]
		IQ	0.014	0.003	0.199	4.89	<0.001	[0.008, 0.020]
		OT	0.034	0.021	0.049	1.62	0.106	[−0.007, 0.075]
		DU	−0.026	0.022	−0.037	−1.18	0.238	[−0.069, 0.017]
		Constant	−0.472	0.204	–	−2.31	0.021	[−0.873, −0.071]

The attenuation of the Group coefficient indicates that measured background characteristics account for part of the initial difference, although a residual association remains. The Practice estimate points in the same direction as the group comparison. These coefficients represent conditional cross-sectional associations and do not isolate change generated by calligraphy practice.

Figure 7 presents *p*-values on a logarithmic scale, allowing the relative statistical evidence for the focal predictors and covariates to be compared across the regression specifications. The Group term in the group-based models and the Practice term in the practice-based model are located below the 0.001 reference level, indicating consistently strong evidence for the principal association with EF. IQ also remains below 0.001 across the adjusted models, showing that general intelligence contributes independently to EF performance after the other listed variables are considered. In contrast, age, gender, education, handedness, other training experience, and digital-device use generally remain above the conventional 0.05 threshold, although their exact p values vary modestly between models.

**FIGURE 7:**
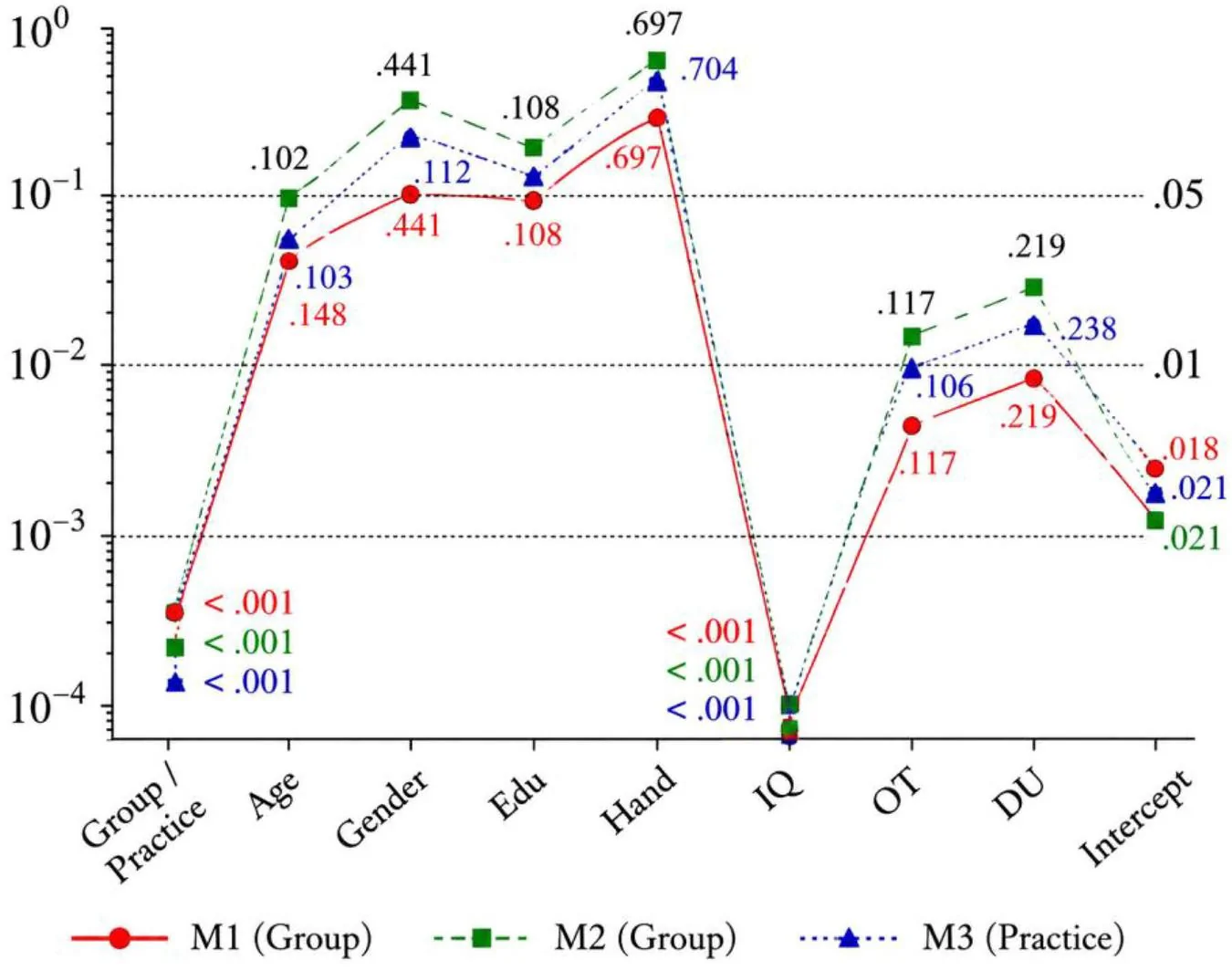
P values across predictors and models.

The transfer models indicate a larger professional–beginner contrast for calligraphy-related tasks than for general cognitive tasks. Table 7 estimates the Group coefficient at 0.548 for near transfer and 0.248 for far transfer, with corresponding p values below 0.001 and 0.007. In the mixed model, the transfer-type coefficient is −0.150 and the Group by transfer-type coefficient is 0.300. This configuration implies that the observed group difference varies by task domain and is more pronounced for near transfer. The far-transfer coefficient remains positive but is comparatively smaller. Because the design is cross-sectional, the interaction is best understood as a difference in conditional group contrasts rather than evidence that practice transferred performance across time.

**TABLE 7:** Models for near- and far-transfer task performance.

Model	Outcome	Predictor	B	SE	β	t/z	P	95% CI
M4	NT	Group	0.548	0.082	0.273	6.68	<0.001	[0.387, 0.709]
		Constant	−0.588	0.242	–	−2.43	0.015	[−1.063, −0.113]
M5	FT	Group	0.248	0.092	0.122	2.7	0.007	[0.067, 0.429]
		Constant	−0.412	0.228	–	−1.81	0.071	[−0.860, 0.036]
M6	Performance	Group	0.248	0.092	–	2.7	0.007	[0.067, 0.429]
		Transfer type	−0.150	0.062	–	−2.42	0.016	[−0.272, −0.028]
		Group × transfer type	0.3	0.083	–	3.61	<0.001	[0.137, 0.463]

### 4.4 Visuospatial structural processing and self-regulation: association models

The indirect-association results are internally consistent across the component paths and the bootstrap estimate. Table 8 reports a Practice–VSP coefficient of 0.297, a VSP–EF coefficient of 0.305, a total Practice–EF coefficient of 0.278, and a direct coefficient of 0.187. The resulting indirect estimate is 0.091, with a bootstrap interval from 0.058 to 0.124 and an indirect proportion of 32.7 percent. The interval does not cross zero, which supports the presence of a statistically identifiable indirect association in this sample. The reduction from the total to the direct coefficient is compatible with VSP accounting for part of the shared variation. Cross-sectional measurement, however, prevents this pattern from establishing temporal sequence.

**TABLE 8:** Indirect-association model through visuospatial structural processing.

Path	Term	B	SE	t	*P*	95% CI low	95% CI high	Boot SE	Boot CI low	Boot CI high
Practice → VSP	a path	0.297	0.039	7.62	<0.001	0.22	0.374	–	–	–
VSP → EF	b path	0.305	0.041	7.44	<0.001	0.224	0.386	–	–	–
Practice → EF	Total (c)	0.278	0.048	5.79	<0.001	0.184	0.372	–	–	–
Practice → EF	Direct (c’)	0.187	0.049	3.82	<0.001	0.091	0.283	–	–	–
Practice → VSP → EF	Indirect	0.091	–	–	–	–	–	0.017	0.058	0.124

Bootstrap resamples = 5,000. The indirect estimate is internally consistent: a × b = 0.0906 and c−c’ = 0.091 (difference due to rounding); Sobel Z = 5.32, *p* < 0.001. The indirect proportion is 32.7%. Given the cross-sectional design, this table reports an indirect association and does not establish temporal ordering or direction.

Figure 8 brings together two complementary visual summaries of the transfer analysis. The left panel plots group performance across near- and far-transfer task types with standard-error bars. The nonparallel profiles and persistent separation between the two groups provide a graphical indication that the professional–beginner contrast differs by transfer domain rather than remaining constant across tasks. This visual pattern corresponds to the mixed-model test in Table 7, in which the Group × transfer-type interaction is statistically significant (B = 0.300, *p* < 0.001). The same model also identifies a transfer-type coefficient of −0.150 (*p* = 0.016) and a positive group coefficient of 0.248 (*p* = 0.007), showing that performance depends jointly on group status and task type. The right panel organizes these relations in a path-style diagram to emphasize how the model components are connected.

**FIGURE 8:**
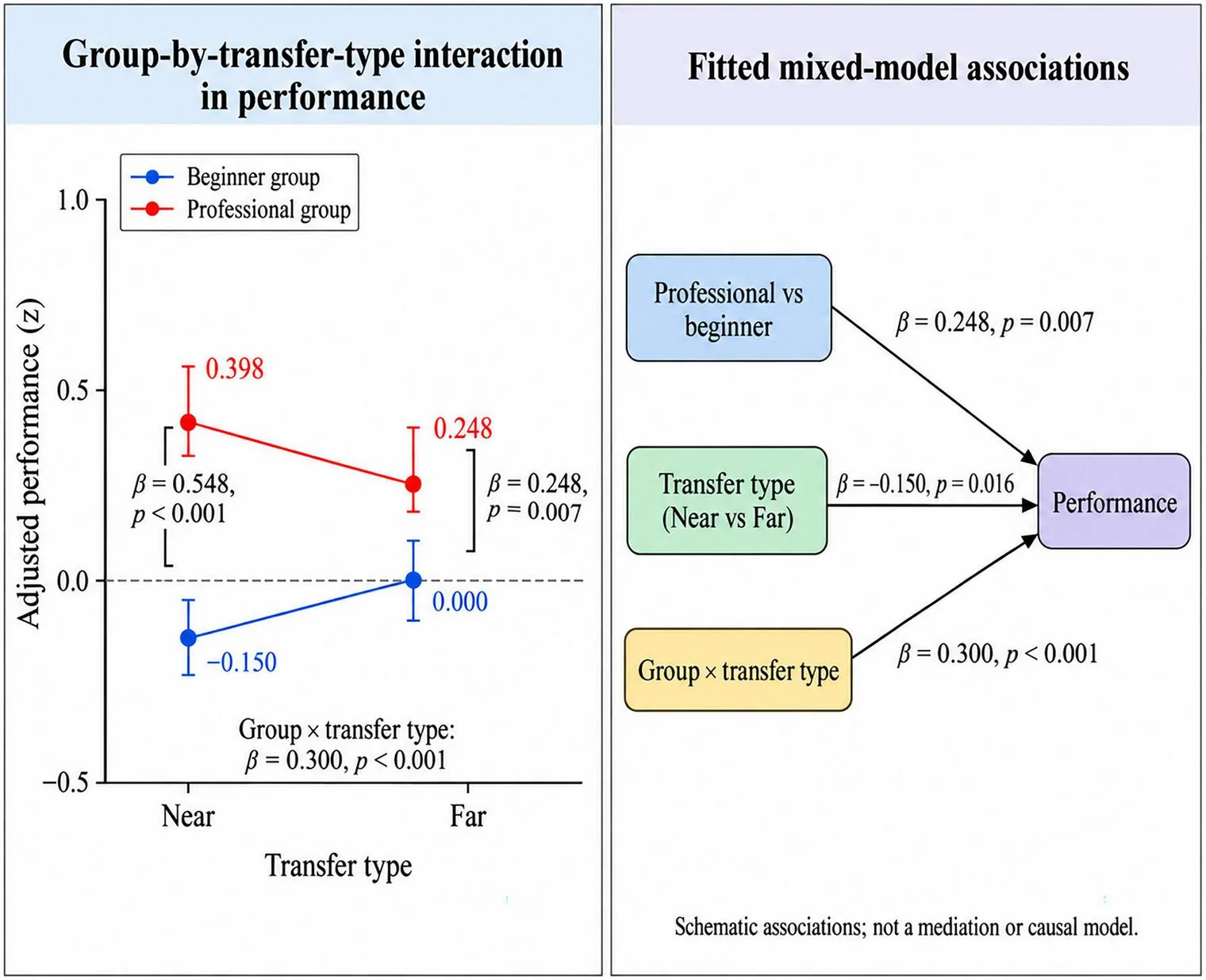
Group-by-transfer-type interaction in performance and a path-style summary of the fitted mixed model. Error bars represent ±1 SE. The adjusted professional–beginner difference was significant for near transfer (*B* = 0.548, *p* < 0.001) and far transfer (*B* = 0.248, *p* = 0.007), and the Group × transfer-type interaction was significant (*B* = 0.300, *p* < 0.001). The right panel is a schematic summary of fitted statistical associations and should not be interpreted as a mediation model or a causal pathway.

Because the study is cross-sectional, the arrows should be read as a schematic representation of fitted statistical associations rather than as a time-ordered mediation mechanism. Taken together, the two panels reinforce the central transfer finding: the magnitude of the observed group difference is task-dependent, and conclusions about near versus far transfer should be based on the interaction estimates and their confidence intervals rather than on a causal interpretation of the diagram.

The moderation model indicates that the Practice–EF association varies with self-regulation after covariate adjustment. Table 9 reports a Practice coefficient of 0.192 with a p value of 0.007, an SR coefficient of 0.152 with a p value of 0.013, and an interaction coefficient of 0.068 with a p value of 0.003. IQ also retains a coefficient of 0.013 with a p value below 0.001. The positive interaction suggests that the conditional Practice slope is larger at higher SR values. This is a statistical pattern rather than evidence that SR amplifies a causal response to practice. Interpretation should therefore focus on heterogeneity in association and on the simple-slope estimates reported separately. Additional model-fit statistics, simpleslope estimates, and fullsample interaction tests are provided in Appendix Tables 3–7.

**TABLE 9:** Moderation analysis of self-regulation.

Variable	M1 B (SE)	t	*P*	M2 B (SE)	t	*P*	M3 B (SE)	t	*P*
Age	0.019 (0.012)	1.64	0.102	0.019 (0.012)	1.63	0.103	0.018 (0.012)	1.55	0.122
Gender (male = 1)	−0.048 (0.062)	−0.77	0.441	−0.047 (0.062)	−0.76	0.448	−0.048 (0.062)	−0.77	0.441
Edu	0.082 (0.051)	1.61	0.108	0.081 (0.051)	1.59	0.112	0.078 (0.051)	1.53	0.127
Hand (right = 1)	0.028 (0.072)	0.39	0.697	0.030 (0.072)	0.42	0.675	0.031 (0.072)	0.43	0.667
IQ	0.014 (0.003)	4.88	<0.001	0.013 (0.003)	4.67	<0.001	0.013 (0.003)	4.62	<0.001
OT	0.033 (0.021)	1.57	0.117	0.034 (0.021)	1.62	0.106	0.035 (0.021)	1.67	0.095
DU	−0.027 (0.022)	−1.23	0.219	−0.026 (0.022)	−1.18	0.238	−0.024 (0.022)	−1.09	0.276
Practice (centered)	–	–	–	0.198 (0.071)	2.79	0.005	0.192 (0.071)	2.7	0.007
SR (centered)	–	–	–	0.156 (0.061)	2.56	0.011	0.152 (0.061)	2.49	0.013
Practice × SR	–	–	–	–	–	–	0.068 (0.023)	2.96	0.003
Constant	−0.485 (0.205)	−2.37	0.018	−0.538 (0.218)	−2.47	0.014	−0.542 (0.218)	−2.49	0.013

### 4.5 Robustness checks

Figure 9 summarizes both effect magnitude and model-explained variance across the main and robustness analyses. In the left panel, group-difference coefficients are generally larger than the corresponding Practice-association coefficients. The largest group coefficient is observed for near transfer (B = 0.548), followed by working memory (0.392), inhibitory control (0.358), cognitive flexibility (0.325), executive attention (0.284), and far transfer (0.248). Practice associations with the EF components are smaller but consistently positive, ranging from 0.178 for executive attention to 0.242 for working memory. The vertical precision axis shows that these estimates differ not only in magnitude but also in uncertainty, so larger coefficients are not automatically the most precisely estimated.

**FIGURE 9:**
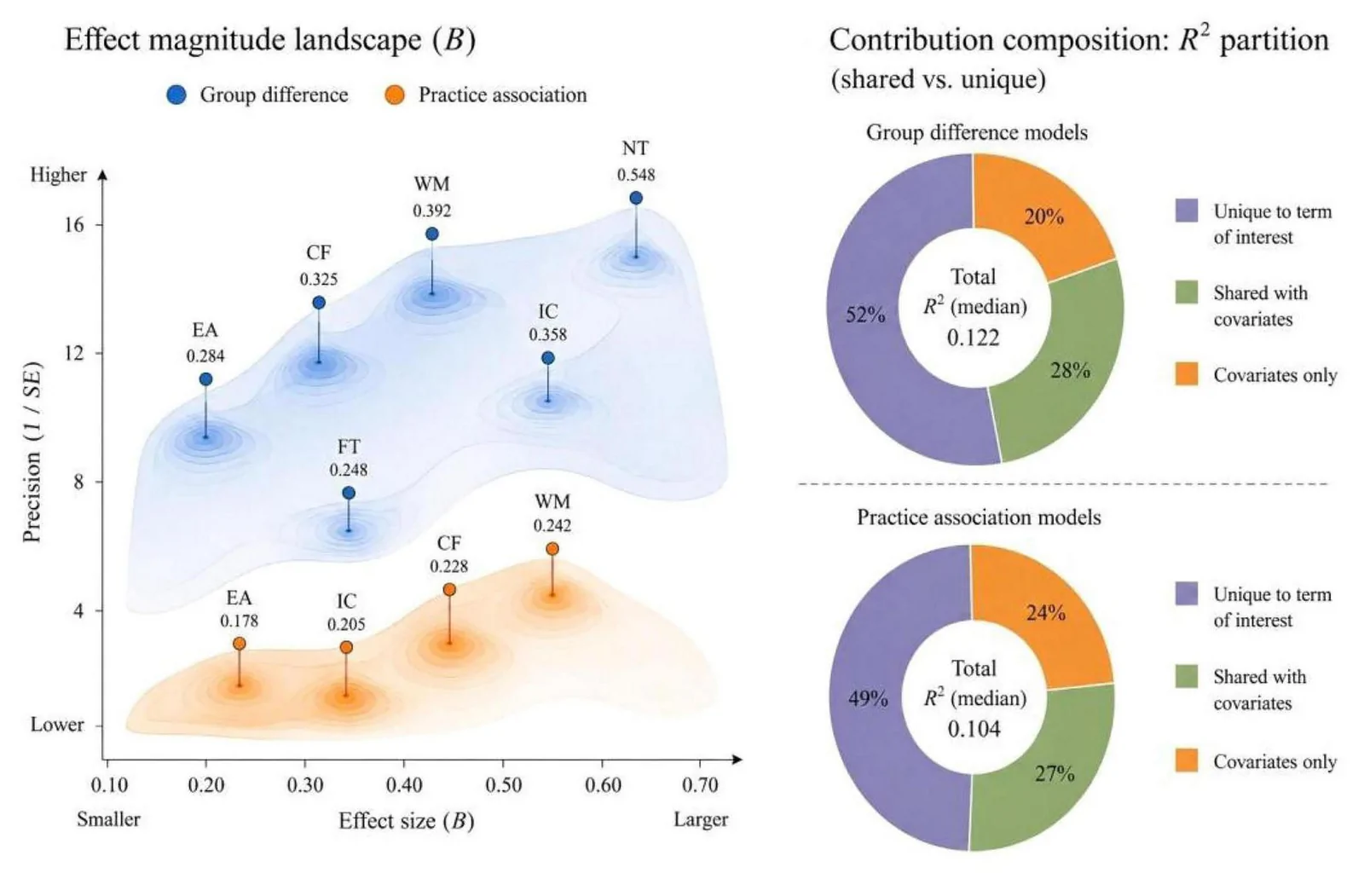
Effect-magnitude landscape plot and variance decomposition (R^2^ partitioning) for group-difference and practice-association models.

The alternative-outcome models show that the main pattern is distributed across several EF components rather than being confined to one score. Table 10 reports Group coefficients of 0.358 for inhibitory control, 0.392 for working memory, 0.325 for cognitive flexibility, and 0.284 for executive attention. The corresponding Practice coefficients are 0.205, 0.242, 0.228, and 0.178. All estimates point in the same positive direction, although their magnitudes vary. Working memory shows the largest coefficient under both specifications, while executive attention shows the smallest. This consistency supports the robustness of the overall EF association, but the component differences should remain descriptive unless formally compared within a common model.

**TABLE 10:** Robustness checks using alternative dependent variables.

Model	Outcome	Term	B	SE	t	*P*	95% CI low	95% CI high	R^2^	F(8, 611)
Group difference	IC	Group	0.358	0.082	4.37	<0.001	0.197	0.519	0.122	10.61
Group difference	WM	Group	0.392	0.088	4.45	<0.001	0.219	0.565	0.138	12.23
Group difference	CF	Group	0.325	0.073	4.45	<0.001	0.182	0.468	0.115	9.93
Group difference	EA	Group	0.284	0.091	3.12	0.002	0.105	0.463	0.092	7.73
Practice association	IC	Practice	0.205	0.075	2.73	0.007	0.058	0.352	0.098	8.3
Practice association	WM	Practice	0.242	0.057	4.25	<0.001	0.13	0.354	0.118	10.22
Practice association	CF	Practice	0.228	0.065	3.51	<0.001	0.1	0.356	0.108	9.25
Practice association	EA	Practice	0.178	0.074	2.41	0.016	0.033	0.323	0.088	7.37
Group difference	NT	Group	0.548	0.082	6.68	<0.001	0.387	0.709	0.178	16.54
Group difference	FT	Group	0.248	0.092	2.7	0.007	0.067	0.429	0.102	8.68

All models in this table models include seven covariates, df = (8, 611). IC, inhibitory control; WM, working memory; CF, cognitive flexibility; EA, executive attention.

Alternative indicators of calligraphy experience yield a broadly consistent relation with EF. Table 11 reports coefficients of 0.286 for years of study, 0.324 for weekly frequency, 0.225 for session duration, 0.267 for experience breadth, and 0.014 for CVS in its original units. The benchmark Practice coefficient is 0.278, with an R-squared of 0.151. Weekly frequency has the largest standardized-indicator coefficient, while session duration has the smallest. The similarity in direction across indicators reduces dependence on a single operational definition of practice. Direct magnitude comparisons with CVS require caution because CVS is not standardized in this table and serves a different validation role.

**TABLE 11:** Robustness checks using alternative practice indicators.

Model	Outcome	Alternative predictor	B	SE	β	t	*P*	95% CI	R^2^	F(8, 611)
R4a	EF	Years of calligraphy study	0.286	0.052	0.278	5.5	<0.001	[0.184, 0.388]	0.128	11.21
R4b	EF	Weekly practice frequency	0.324	0.061	0.316	5.31	0.001	[0.204, 0.444]	0.137	12.13
R4c	EF	Practice duration per session	0.225	0.052	0.219	4.33	0.001	[0.123, 0.327]	0.108	9.25
R4d	EF	Breadth of calligraphy experience	0.267	0.062	0.26	4.31	0.001	[0.145, 0.389]	0.117	10.13
R4e	EF	CVS	0.014	0.002	0.291	5.97	0.001	[0.009, 0.019]	0.138	12.23
R4f	EF	Practice (benchmark)	0.278	0.048	0.352	5.79	0.001	[0.184, 0.372]	0.151	13.6

All models include the seven covariates, df = (8, 611). R4f is identical to M3. Alternative predictors were standardized except CVS, which is reported in its original units.

The Practice coefficient remains stable as covariates and nonlinear terms are introduced. Table 12 reports estimates of 0.277 without covariates, 0.276 after age and gender, 0.272 after education and handedness, 0.263 after IQ, and 0.278 in the full model. Adding IQ squared yields 0.275, while adding Practice squared yields 0.279. The narrow coefficient range indicates that the observed Practice–EF association is not highly sensitive to these specification changes. The temporary reduction after IQ enters the model is consistent with shared variation between general intelligence and EF. Stability across specifications strengthens robustness, although it cannot eliminate bias from unmeasured characteristics.

**TABLE 12:** Covariate sensitivity analyses.

Model	Covariate set	B	SE	β	t	*P*	95% CI	R^2^	F	df
R5a	No covariates	0.277	0.03	0.35	9.29	<0.001	[0.218, 0.335]	0.123	86.3	(1, 618)
R5b	+Age + Gender	0.276	0.031	0.349	8.9	<0.001	[0.215, 0.337]	0.126	29.6	(3, 616)
R5c	+Edu + Hand	0.272	0.031	0.344	8.77	<0.001	[0.211, 0.333]	0.131	18.5	(5, 614)
R5d	+ IQ	0.263	0.031	0.333	8.48	<0.001	[0.202, 0.324]	0.148	17.7	(6, 613)
R5e	+OT + DU (full model)	0.278	0.048	0.352	5.79	<0.001	[0.184, 0.372]	0.151	13.6	(8, 611)
R5f	Full + IQ^2^	0.275	0.049	0.348	5.61	<0.001	[0.179, 0.371]	0.152	12.1	(9, 610)
R5g	Full + Practice^2^	0.279	0.049	0.353	5.69	<0.001	[0.183, 0.375]	0.151	12	(9, 610)

R5a–R5e form a nested sequence; R^2^ increases from 0.123 to 0.151. R5f and R5g are parallel extensions of R5e. For R5a, t = 9.29 and F = t^2^ = 86.3 based on the unrounded simple correlation; IQ^2^ (*p* = 0.48) and Practice^2^ (*p* = 0.55) are not statistically significant.

Figure 10 provides a visual robustness profile for the Practice–EF coefficient under alternative approaches to influential observations. The benchmark estimate is B = 0.278 with SE = 0.048 and a 95% confidence interval from 0.184 to 0.372. One-percent and five-percent two-sided Winsorization produce slightly smaller coefficients of 0.271 and 0.263, whereas excluding observations with | Z| > 3 or | Z| > 2.5 yields somewhat larger coefficients of 0.286 and 0.294. Median regression returns an estimate of 0.271.

**FIGURE 10:**
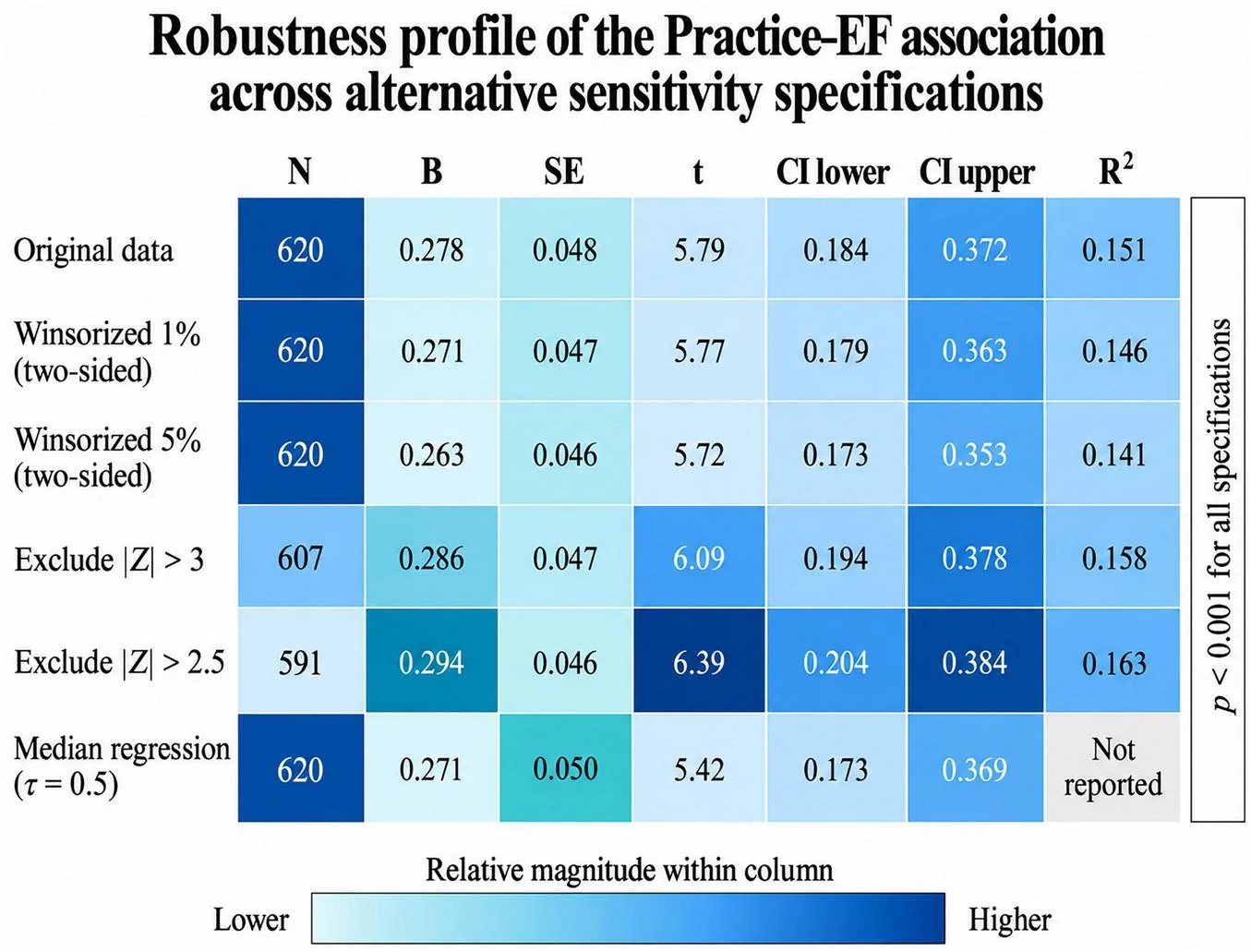
Robustness profile of the treatment effect on IQ across alternative sensitivity specifications.

## 5 Discussion

### 5.1 Main findings and theoretical contributions

This study examined cross-sectional associations among calligraphy practice, executive function, near transfer, and far transfer, together with VSP and SR. Professionals had higher adjusted EF, near-transfer, and far-transfer scores than beginners, and the group difference was larger for near than far transfer. Practice was positively associated with EF. The VSP model yielded a statistically significant indirect association, while the Practice × SR term indicated heterogeneity in the conditional association. These results extend prior work by jointly modeling group differences, transfer-type differences, and association pathways, but they do not show that calligraphy practice produced the observed cognitive differences.

The comparative synthesis places the current findings within several adjacent research traditions while preserving the study’s observational scope. Table 13 summarizes the comparative context and the balanced sample of 310 participants per group, while Tables 6–8 report the adjusted Group–EF coefficient of 0.415, the Group–NT and Group–FT coefficients of 0.548 and 0.248, the Group × transfer-type interaction coefficient of 0.300, and the bootstrap interval of 0.058 to 0.124 for the indirect association. Together, these estimates show that the largest adjusted group contrast occurs for near transfer, while the far-transfer contrast is smaller. The comparison supports an association-focused contribution across calligraphy, executive-function, and transfer research, without implying that the cross-sectional design identifies cognitive change.

**TABLE 13:** Comparison of empirical results between previous studies and this study.

Type of study	Sample and task design	Key concerns	Main result characteristics
Research on executive function training	Short term Stroop, MSIT or anti saccade task training was used. The training cycle of some studies was 5–7 days	Conflict control, executive attention, and transfer patterns	Within-task improvement is usually larger; transfer varies across task and design conditions.
Artificial-grammar learning and transfer research	Study transfer through rule learning, surface structure change and transfer judgment task	Association of learned rules with new materials or structures	Transfer patterns vary with shared structure, block information, and rule abstraction.
Research on mathematical cognition and executive function	Involving natural number knowledge, fractional knowledge, working memory, inhibition control and cognitive flexibility	The role of executive function in concept integration, strategy selection and interference control	There is a differential relationship between different executive function components and learning tasks.
Calligraphy education and calligrapher research	Pay attention to copying, specialize in one family, turn to many teachers, self-evaluation and cultural cultivation.	The formation of calligraphy skills and the growth path of Calligraphers	Emphasize the importance of long-term practice, structural understanding, self-education and aesthetic experience
This study	620 valid samples, 310 for professional calligraphers and 310 for beginners	Calligraphy practice, EF, near transfer, far transfer, indirect association, and statistical interaction	Adjusted cross-sectional differences and associations; no causal inference.

### 5.2 Enlightenment from educational practice

The findings should not be used to claim that calligraphy instruction improves executive function. They can, however, motivate testable educational hypotheses. Calligraphy courses may consider emphasizing observation, comparison, structural analysis, error checking, and reflective goal setting, while any cognitive benefit should be evaluated in preregistered longitudinal or randomized studies. For beginners, font-structure judgment, spatial-proportion discrimination, and stroke-direction recognition may be incorporated as calligraphy-learning activities without presenting them as proven cognitive interventions.

### 5.3 Research limitations

This study has several limitations. First, the cross-sectional comparison identifies group differences and associations but cannot determine temporal order, rule out self-selection, or support causal claims. Second, professional calligraphers may differ in script preference, training pathway, teacher guidance, motivation, socioeconomic background, and creative experience; measured covariates do not eliminate residual confounding. Third, task versions, trial inventories, timing parameters, acquisition software, and item-level source identifiers must be documented from archived scripts, and CVS, NT, VSP, EF, and FT must be confirmed to use mutually exclusive items, trials, and score columns. Any detected overlap requires recomputation of the affected composites and all dependent analyses. Fourth, behavioral measures were not complemented by eye tracking, writing trajectories, or neurophysiological data. Finally, the predominantly student sample limits generalizability across age and educational groups.

### 5.4 Future outlook

Future research should use longitudinal tracking or randomized calligraphy-training designs to establish temporal ordering and test whether within-person cognitive change differs from an appropriate control condition. Studies may compare regular, running, and cursive script experience; preregister near-transfer and far-transfer outcomes; use independent task batteries; and report complete trial-level protocols and analysis code. Eye tracking, writing trajectories, pressure sensing, or EEG may help evaluate proposed explanations, while broader age and educational samples would clarify generalizability.

## 6 Conclusion

This cross-sectional study found that professional calligraphers and beginners differed in EF, near-transfer, and far-transfer performance, and that calligraphy-practice indicators were associated with EF after covariate adjustment. The larger group difference for near than far transfer is consistent with task-similarity accounts. VSP showed an indirect statistical association and SR formed a significant statistical interaction with Practice. These findings provide an association-focused framework for studying calligraphy experience and cognition, but they do not establish temporal ordering or within-person improvement. Longitudinal and experimental evidence is required before directional or educational-benefit claims can be made.

## Data Availability

The raw data supporting the conclusions of this article will be made available by the author, without undue reservation.

## Appendix

**APPENDIX 1:** Task implementation details requiring confirmation from archived scripts.

Construct	Task/version and platform	Trials and stimuli	Timing parameters	Scoring
CVS	Prescribed copying + font-structure judgment + expert blind rating. Local task revision, rater number, blinding procedure, and platform: AUTHOR TO VERIFY.	Exact copying-item and judgment-trial counts; character list and item IDs: AUTHOR TO VERIFY.	Presentation, response window, ITI, and break schedule: AUTHOR TO VERIFY.	Report component ranges, standardization/weights, expert ICC, and software. CVS is post-group validation only.
Near transfer (NT)	Font-structure judgment, stroke-direction conflict, spatial-proportion recognition, and calligraphic-structure recognition. Versions/platform: AUTHOR TO VERIFY.	Exact trials per subtask and unique stimulus/item IDs: AUTHOR TO VERIFY.	Fixation, stimulus duration, response window, ITI, and blocks: AUTHOR TO VERIFY.	Report accuracy/RT transformation, subtask weights, reliability, and composite formula.
Far transfer (FT)	Stroop, Flanker, n-back, and task-switching/attention tasks. Exact variants, n-back load, versions, and platform: AUTHOR TO VERIFY.	Congruent/incongruent ratios, target/non-target ratios, blocks, and total unique trials: AUTHOR TO VERIFY.	Fixation, stimulus duration, response window, ITI, feedback, and breaks: AUTHOR TO VERIFY.	Report task-specific contrast formulas, direction reversal, standardization, reliability, and composite formula.
VSP	A separately administered visuospatial task with no CVS or NT items. Actual instrument/version and platform: AUTHOR TO VERIFY.	Unique non-reused stimuli, practice trials, test trials, and item IDs: AUTHOR TO VERIFY.	Presentation, response window, ITI, and block structure: AUTHOR TO VERIFY.	Report accuracy/RT rule, standardization, reliability, and final VSP formula.
EF	Four independent source tasks for inhibitory control, working memory, cognitive flexibility, and executive attention. Exact task names/versions/platform: AUTHOR TO VERIFY.	Practice/test trials and source-column IDs for IC, WM, CF, and EA: AUTHOR TO VERIFY.	Fixation, stimulus duration, response window, ITI, and breaks: AUTHOR TO VERIFY.	Report each component formula, direction reversal, z-standardization, weights, reliability, and EF composite formula.
SR	Self-regulation scale name, version, language adaptation, and administration mode: AUTHOR TO VERIFY.	Number of items, response anchors, reverse-coded items, and missing-item rule: AUTHOR TO VERIFY.	Self-paced; actual administration window: AUTHOR TO VERIFY.	Report sum/mean rule, possible range, Cronbach’s α/ω, and software.

**APPENDIX 2:** Construct-separation audit and reanalysis decision rule.

Comparison	Required separation	Evidence currently available	Status	Required action
Practice vs. CVS	CVS score excluded from the Practice composite	Current variable specification in Table 2	Separated at score-definition level	Retain; add code/column IDs to supplement
CVS vs. NT	No shared characters, items, judgment trials, or derived scores	Conceptual descriptions only; no item IDs	Not yet demonstrated	Audit scripts/logs and report disjoint item IDs
NT vs. VSP	No shared stimuli, trials, or score columns	Conceptual descriptions only; no source IDs	Not yet demonstrated	Audit; use an independent VSP task or recompute
EF vs. FT	No EF component task score reused in FT	Current descriptions name potentially overlapping task families	High-priority audit	Assign independent source tasks; recompute all affected analyses if overlap exists
All composites	Each source column belongs to one construct only	No item-to-composite crosswalk supplied	Not yet demonstrated	Provide a source-column crosswalk and reproducible scoring code

**APPENDIX 3:** Model fit.

Model	Predictors	df	R^2^	Adjusted R^2^	F
M1	1	(1, 618)	0.165	0.164	122.1
M2	8	(8, 611)	0.148	0.137	13.27
M3	8	(8, 611)	0.151	0.14	13.6

M3 is EF∼Practice + Age + Gender + Edu + Hand + IQ + OT + DU; R^2^ = 0.151 is the common benchmark used in the robustness tables.

**APPENDIX 4:** Model fit.

Model	Predictors	df	R^2^	Adjusted R^2^	F/Wald
M4	8	(8, 611)	0.178	0.167	16.54
M5	8	(8, 611)	0.102	0.09	8.68
M6	10	–	0.215	–	Wald = 82.4

M4 and M5 include the seven covariates shown in M2. M6 is a mixed-effects model. Group: professional, 1; beginner, 0; transfer type: near, 1; far, 0. The model-implied near-transfer group difference is 0.248 + 0.300 = 0.548.

**APPENDIX 5:** Model fit.

Metric	M1	M2	M3
df	(7, 612)	(9, 610)	(10, 609)
R^2^	0.128	0.168	0.18
Adjusted R^2^	0.118	0.156	0.167
ΔR^2^	–	0.040***	0.012**
F	12.83***	13.69***	13.37***

***p* < 0.01, ****p* < 0.001.

**APPENDIX 6:** Simple slopes derived from the Model 3 variance–covariance matrix.

SR level	SR value	Slope formula	B	SE	t	*P*	95% CI
Low (−1 SD)	3.464	0.192–0.068 × 1.186	0.111	0.088	1.26	0.208	[−0.062, 0.284]
Mean	4.65	0.192	0.192	0.071	2.7	0.007	[0.053, 0.331]
High (+1 SD)	5.836	0.192 + 0.068 × 1.186	0.273	0.062	4.4	<0.001	[0.151, 0.395]

Cov(BPractice, BPractice × SR) = −0.0008. The mean-SR slope equals the Model 3 Practice coefficient. The low-SR slope is not statistically different from zero; the interaction indicates that the conditional Practice–EF association varies with SR.

**APPENDIX 7:** Full-sample interaction tests.

Interaction	B	SE	t	*P*
Practice × gender	−0.032	0.067	−0.48	0.631
Practice × age group	0.046	0.066	0.7	0.484
Practice × IQ group	0.077	0.065	1.18	0.238

The full-sample row is identical to M3. Each subsample model controls the covariates other than the stratifying variable. The interaction terms test whether the Practice coefficient differs across strata.
